# Metabolic control of ferroptosis in cancer: lipid dependencies as therapeutic vulnerabilities

**DOI:** 10.3389/fonc.2026.1872103

**Published:** 2026-07-23

**Authors:** Anna Di Dio, Gerardina Smaldone, Valeria Napolitano, Alessia Bertamino, Isabel M. Gomez-Monterrey

**Affiliations:** 1 Department of Pharmacy, University of Salerno, Fisciano, Italy; 2 Department of Pharmacy, University of Naples Federico II, Naples, Italy

**Keywords:** ACSL4, drug-tolerant persister cells, ferroptosis, FSP1, GPx4, lipid metabolism, lipid peroxidation, MBOAT1/2

## Abstract

Ferroptosis is an iron-dependent form of regulated cell death driven by the accumulation of peroxidized phospholipids in cellular membranes. In cancer, susceptibility to ferroptosis is not fixed but instead reflects a dynamic metabolic state shaped by lipid remodeling programs that determine membrane composition, oxidative liability, and the capacity to detoxify lipid peroxides. Tumor cells rewire fatty acid synthesis, desaturation, esterification, storage, sterol metabolism, and ether phospholipid remodeling to alter the abundance and distribution of oxidizable phospholipids and thereby shift their ferroptotic threshold. Polyunsaturated fatty acid-rich membrane phospholipids and di-polyunsaturated phospholipids promote lipid peroxidation and ferroptosis sensitivity, whereas monounsaturated fatty acids, lipid-droplet sequestration, 7-dehydrocholesterol, membrane-bound O-acyltransferase domain-containing 1 and 2, and antioxidant defense systems including glutathione peroxidase 4, ferroptosis suppressor protein 1, and the GTP cyclohydrolase 1-tetrahydrobiopterin pathway suppress ferroptotic death. Therapy-resistant, mesenchymal-like, and drug-tolerant persister states often display elevated oxidative stress together with increased dependence on lipid peroxide detoxification, whereas cancer stem cell-like states can remain either buffered or vulnerable depending on context. Here, we synthesize how lipid-state remodeling, tumor genotype, cell-state plasticity, and microenvironmental cues position tumors along a functional ferroptotic threshold, and we discuss how integrated lipidomic, transcriptional, and state-associated biomarkers may support biomarker-guided ferroptosis-based strategies in precision oncology.

## 1 Introduction

Ferroptosis is a regulated form of cell death characterized by iron-dependent lipid peroxidation and the accumulation of oxidized phospholipids within cellular membranes. Unlike apoptosis and necroptosis, ferroptosis is fundamentally metabolic in origin, linking cell fate directly to membrane lipid composition, redox balance, and iron homeostasis (1–4).

In cancer, ferroptosis can act both as a tumor-suppressive mechanism and as a therapeutically exploitable vulnerability. Tumor cells commonly operate under heightened oxidative stress because of oncogenic signaling and metabolic rewiring while simultaneously activating compensatory programs that enhance antioxidant capacity and remodel membrane lipids. These adaptations support survival under oxidative pressure, but they also create context-dependent metabolic dependencies that may be selectively targeted (2, 4).

Among the determinants of ferroptosis sensitivity, lipid metabolism is especially important because it reshapes membrane phospholipid composition and thereby determines susceptibility to lipid peroxidation. Cancer cells dynamically regulate fatty acid synthesis, desaturation, esterification, storage, and sterol metabolism to tune the abundance and distribution of oxidizable phospholipids. Polyunsaturated lipid enrichment generally promotes ferroptosis, whereas monounsaturated fatty acid incorporation, sequestration of polyunsaturated lipids into neutral lipid stores, and activation of antioxidant defenses suppress it (3).

Recent reviews have comprehensively summarized the core cell biology of ferroptosis and its regulation by lipid metabolism (2–4). Rather than recapitulating those broader overviews, the present review asks a more specific cancer-metabolism question: how do lipid remodeling programs, tumor plasticity, lineage context, and antioxidant redundancy combine to generate therapeutic vulnerabilities that can be recognized and exploited in oncology? This emphasis allows us to integrate recent lipid biology, including peroxisome-derived ether phospholipids, 7-dehydrocholesterol metabolism, and membrane-bound O-acyltransferase domain-containing 1 and 2-mediated phospholipid remodeling, into a cancer-focused and biomarker-aware framework (5–10).

**Figure 1:**
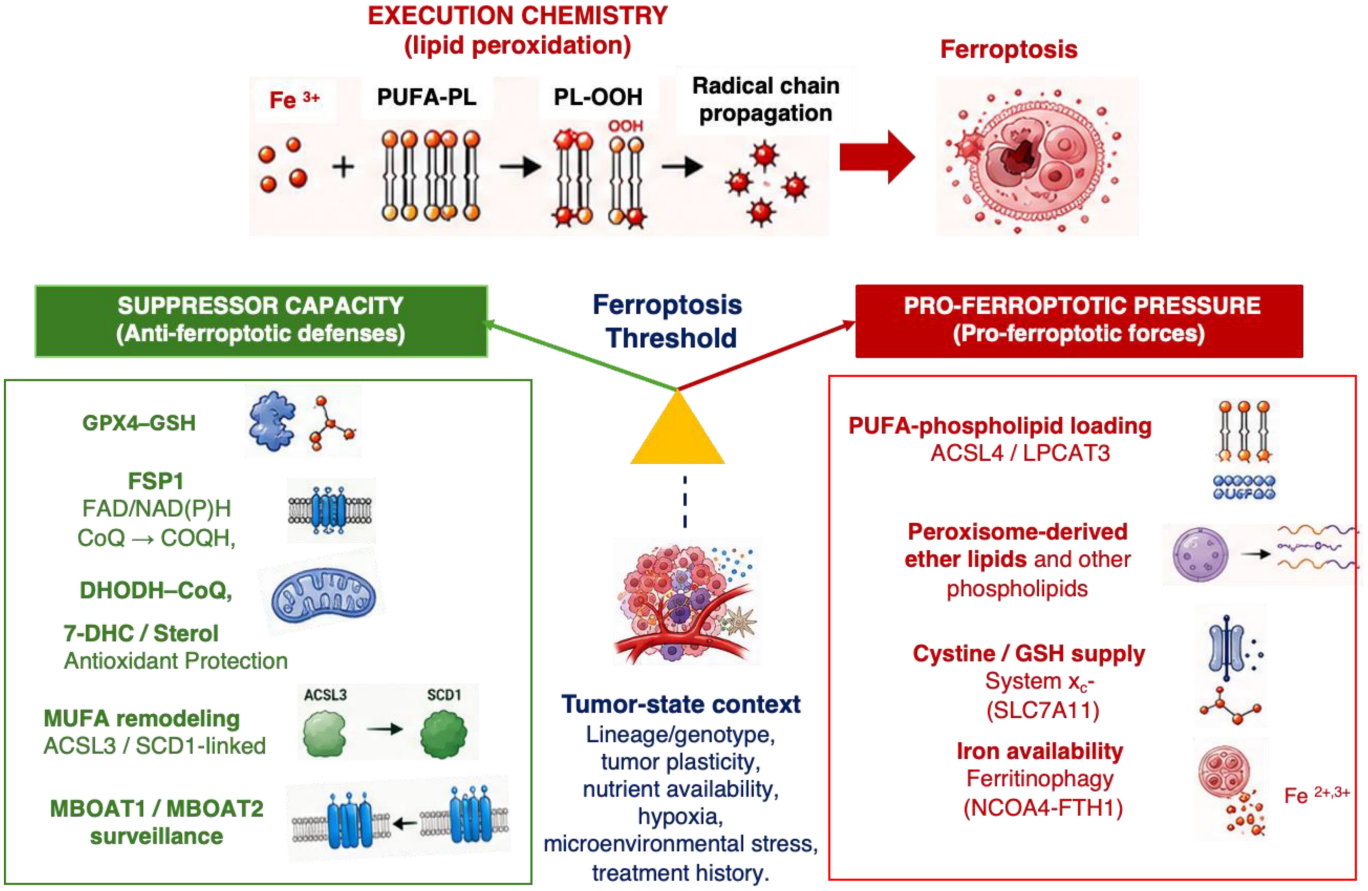
Determinants of ferroptosis susceptibility in cancer. Ferroptosis sensitivity is defined by the balance between pro-ferroptotic lipid peroxidation pressure and anti-ferroptotic antioxidant buffering, which together establish a functional ferroptotic threshold. Tumor lineage/genotype, microenvironmental inputs, and adaptive cell states modulate this threshold. Anti-ferroptotic buffering includes the GPX4–glutathione axis, system xC−/SLC7A11-dependent cystine utilization, FSP1–CoQ and GCH1–BH4 defense systems, mitochondrial CoQ reduction, monounsaturated fatty acid synthesis, lipid droplet sequestration, 7-dehydrocholesterol, and suppressive phospholipid remodeling pathways. Pro-ferroptotic lipid pressure includes ACSL4/LPCAT3-dependent PUFA incorporation, oxidized phosphatidylethanolamine species, di-polyunsaturated phospholipids, ether-lipid remodeling linked to peroxisomal metabolism, iron and reactive oxygen species, and mesenchymal-like or drug-tolerant states. Together, these determinants position tumor cells along a continuum from ferroptosis-resistant to ferroptosis-sensitive states.

Ferroptosis susceptibility is therefore best understood as a functional threshold set by the balance between lipid peroxidation pressure and the buffering capacity of antioxidant defense systems. Position along this threshold is shaped not only by tumor-intrinsic signaling but also by tumor cell state, nutrient availability, and microenvironmental stress. As summarized in **Figure 1**, tumor lipid architecture, antioxidant buffering, lineage/genotype, and adaptive plasticity collectively determine where a tumor sits on this threshold.

## 2 Lipid reprogramming and tumor-state determinants of ferroptosis vulnerability

In cancer, ferroptosis susceptibility emerges from the interaction of membrane lipid composition, redox homeostasis, oncogenic signaling, metabolic context, and cellular state. At the center of this vulnerability lies lipid reprogramming, which determines the abundance, identity, and subcellular distribution of oxidizable phospholipid species. Rather than arising from a single molecular defect, ferroptosis in tumors reflects how these processes converge on a shared liability: the accumulation of phospholipid hydroperoxides beyond the detoxification capacity of antioxidant defense systems (1, 11, 12).

Selective incorporation of omega-6 polyunsaturated fatty acids into membrane phospholipids is a major determinant of ferroptosis sensitivity. Acyl-CoA synthetase long-chain family member 4 (ACSL4) preferentially activates arachidonic acid and adrenic acid, which are subsequently incorporated into phosphatidylethanolamine species by lysophosphatidylcholine acyltransferase 3 (LPCAT3), thereby generating phospholipids that are highly susceptible to peroxidation (13–15). Beyond increasing the abundance of oxidizable phospholipids, ferroptosis execution also involves regulated site-specific oxidation events, including the generation of oxidized phosphatidylethanolamines by lipoxygenase (LOX)-phosphatidylethanolamine-binding protein 1 (PEBP1) complexes that function as death-promoting signals (16–18). Recent lipidomic studies have further highlighted phospholipids carrying two polyunsaturated acyl tails, often referred to as di-polyunsaturated phospholipids, as potent amplifiers of ferroptosis that accelerate radical propagation and lower the functional threshold for cell death (19, 20). Spatial lipidomics has likewise pointed to the endoplasmic reticulum as a critical site of peroxidation initiation, while protein kinase C beta II-dependent phosphorylation of ACSL4 can further amplify polyunsaturated phospholipid incorporation (21, 22).

Peroxisome- and ether-lipid biology has expanded this framework in important ways. Peroxisome-derived polyunsaturated ether phospholipids (PUFA-ePLs) provide highly oxidizable substrates for ferroptosis, and their synthesis depends on enzymes such as alkylglycerone phosphate synthase (AGPS) and fatty acyl-CoA reductase 1 (FAR1) within the ether-lipid biosynthetic pathway (5). Complementary work showed that a FAR1-transmembrane protein 189 (TMEM189; also known as plasmanylethanolamine desaturase 1) axis shapes alkyl-ether lipids and plasmalogens and can modulate ferroptosis sensitivity in a context-dependent manner (6). Together, these studies indicate that ether-phospholipid composition is not peripheral to ferroptosis control; rather, it is a dynamic determinant of how strongly membranes support or resist lipid-peroxide propagation. Importantly, downregulation of PUFA-ePLs can accompany acquired ferroptosis resistance, highlighting lipid remodeling as an adaptive process during tumor evolution (5).

Tumor cells can also suppress ferroptosis through anti-ferroptotic lipid remodeling. Activation of the phosphoinositide 3-kinase-protein kinase B-mechanistic target of rapamycin (PI3K-AKT-mTOR) pathway promotes sterol regulatory element-binding protein-dependent lipogenesis, thereby favoring the synthesis of saturated and monounsaturated fatty acids (23, 24). Monounsaturated fatty acid incorporation reduces susceptibility to lipid peroxidation by lowering bis-allylic bond density, and excess polyunsaturated fatty acids can be redirected into triglycerides and sequestered within lipid droplets through diacylglycerol acyltransferase 1/2 (DGAT1/2)-dependent pathways (25–28). Two recently appreciated suppressive mechanisms deserve particular attention. First, membrane-bound O-acyltransferase domain-containing 1 and 2 (MBOAT1/2) independently suppress ferroptosis through phospholipid remodeling without requiring glutathione peroxidase 4 (GPX4) or ferroptosis suppressor protein 1 (FSP1), and their expression is linked to estrogen- and androgen-receptor signaling, respectively (7). Second, distal cholesterol biosynthesis can generate the sterol intermediate 7-dehydrocholesterol (7-DHC), which acts as a radical-trapping metabolite that shields membranes from autoxidation and suppresses ferroptosis (8, 9). These findings reinforce the idea that ferroptosis resistance can be lipid-encoded, not enzyme-encoded.

The ferroptotic threshold is also governed by the efficiency and redundancy of antioxidant systems. GPX4, supported by cystine import through the cystine/glutamate antiporter system xC− and glutathione synthesis, is the principal defense against phospholipid hydroperoxide accumulation (11, 29). Complementary defense systems include the FSP1-coenzyme Q (CoQ) axis and the GTP cyclohydrolase 1-tetrahydrobiopterin (GCH1-BH4) pathway, both of which suppress lipid peroxidation independently of GPX4 (30–32). Mitochondrial pathways, including dihydroorotate dehydrogenase (DHODH)-dependent CoQ reduction, and broader determinants of nicotinamide adenine dinucleotide phosphate (reduced form; NADPH) availability likewise contribute to redox homeostasis (33, 34). Accordingly, tumors with impaired NADPH regeneration or restricted cystine availability are often more vulnerable to ferroptosis.

These metabolic programs are coordinated by oncogenic signaling pathways. Activation of the RAS-RAF-mitogen-activated protein kinase axis can enhance reactive oxygen species (ROS) production and promote synthesis of polyunsaturated phospholipids, thereby increasing ferroptosis susceptibility in selected contexts (12, 35). By contrast, activation of the Kelch-like ECH-associated protein 1-nuclear factor erythroid 2-related factor 2 (KEAP1-NRF2) pathway induces detoxification programs that enhance cystine utilization, glutathione synthesis, and lipid-peroxide buffering, thereby conferring ferroptosis resistance (36–38). Tumor protein p53 (TP53) can either facilitate or restrain ferroptosis depending on context, in part through regulation of solute carrier family 7 member 11 (SLC7A11) and related metabolic pathways (39, 40). Dependence on glutaminolysis, oxidative phosphorylation, or fatty acid desaturation can further expand pools of oxidizable lipids, whereas nutrient stress—including cystine restriction—can expose latent ferroptotic liabilities (35, 41–44).

Cellular state and plasticity are equally important. Drug-tolerant persister cells and mesenchymal-like tumor states frequently display elevated oxidative stress together with increased reliance on lipid peroxide detoxification pathways, rendering them particularly vulnerable to ferroptosis induction (13, 45, 46). Clear-cell carcinomas represent another clinically relevant GPX4-dependent state, in which hypoxia-inducible factor-linked polyunsaturated lipid enrichment underlies intrinsic ferroptosis sensitivity (47). Cancer stem cell-like states, by contrast, are not uniformly ferroptosis-sensitive; in some settings they couple enhanced redox buffering, SLC7A11 expression, and adaptive lipid remodeling to relative resistance, although this remains context dependent (48). Autophagy-related processes, including ferritinophagy and lipophagy, also influence ferroptosis sensitivity by regulating intracellular iron availability and lipid substrate mobilization, respectively (27, 49). Ferroptosis further intersects with tumor immunity: activated cluster of differentiation 8-positive (CD8+) T cells can promote ferroptosis in tumor cells through interferon-gamma-mediated repression of SLC7A11, whereas GPX4 remains essential for survival and function in several immune subsets, including regulatory T cells (50–52). Together, these findings indicate that ferroptosis sensitivity is determined not only by tumor-intrinsic metabolism but also by immune and microenvironmental interactions.

## 3 Ferroptosis-targeted therapeutic strategies

The determinants of ferroptosis sensitivity outlined above provide a conceptual basis for therapeutic intervention. Tumor-specific lipid remodeling, antioxidant adaptation, and redox stress create exploitable vulnerabilities within the ferroptosis regulatory network. In practice, however, ferroptosis-targeted therapies are unlikely to be uniformly effective across cancers and may be most useful when matched to specific lipid and antioxidant dependencies.

Current therapeutic strategies broadly fall into two categories: those that increase lipid peroxidation pressure and those that disable antioxidant defenses (**Table 1**). Viewed through the framework of ferroptotic threshold control, both approaches aim to shift tumor cells from buffered survival toward irreversible oxidative collapse. Among antioxidant-directed approaches, direct targeting of GPX4 remains central. Covalent GPX4 inhibitors and GPX4 degradation strategies prevent detoxification of phospholipid hydroperoxides and thereby trigger ferroptotic cell death (11, 45, 53–59). Although most GPX4-directed compounds remain preclinical, they have established proof of concept across multiple cancer models.

**Table 1:** Pharmacological and emerging lipid-remodeling strategies targeting the ferroptosis regulatory network.

Functional axis	Target pathway	Representative agents/approaches	Mechanistic effect	Development stage	Key references
Inhibition of antioxidant defenses	GPX4	RSL3, ML162, ML210, JKE-1674	Blocks phospholipid hydroperoxide detoxification and increases lipid peroxidation	Preclinical	(11, 45, 54–56)
Inhibition of antioxidant defenses	GPX4 degradation	PROTAC-based degraders	Promotes proteasomal GPX4 loss and sustains ferroptotic stress	Early preclinical	(57–59)
Inhibition of antioxidant defenses	system xC−/SLC7A11	Erastin, IKE, sulfasalazine, sorafenib	Reduces cystine uptake, depletes glutathione, and indirectly impairs GPX4 activity	Preclinical/translational	(41, 53, 60–62)
Inhibition of antioxidant defenses	Glutathione synthesis	BSO, APR-246	Depletes intracellular glutathione and weakens antioxidant buffering	Translational	(61, 62)
Inhibition of antioxidant defenses	FSP1-CoQ pathway	Experimental FSP1 inhibitors	Blocks GPX4-independent lipid-radical detoxification	Preclinical	(30, 63, 64)
Inhibition of antioxidant defenses	Combined antioxidant defense targeting	GPX4 plus FSP1 inhibition	Prevents compensatory resistance and maximizes lipid-peroxide accumulation	Emerging preclinical rationale	(30, 65)
Amplification of lipid peroxidation	GPX4 destabilization/CoQ depletion	FIN56	Promotes GPX4 degradation and CoQ depletion	Preclinical	(66, 67)
Amplification of lipid peroxidation	Iron-dependent oxidation	FINO2	Promotes iron-mediated lipid oxidation and ROS-driven peroxidation	Preclinical	(68)
Emerging lipid-remodeling targets	MBOAT1/MBOAT2	Hormone blockade plus ferroptosis induction	Disrupts GPX4-independent phospholipid remodeling-based ferroptosis surveillance	Emerging preclinical rationale	(7)
Emerging lipid-remodeling targets	Distal cholesterol biosynthesis/7-DHC axis	Context-matched modulation of DHCR7/EBP/7-DHC	Alters 7-DHC-mediated radical trapping and ferroptosis sensitivity in a context-dependent manner	Emerging preclinical rationale	(8–10)

Therapeutic utility is expected to depend on tumor lipid composition, antioxidant redundancy, and the dominant ferroptosis-defense program rather than on a universal ferroptosis-sensitive phenotype.

Complementary strategies act upstream of GPX4 by limiting cystine availability or glutathione synthesis. Inhibition of system xC− through blockade of SLC7A11 reduces intracellular cystine pools, depletes glutathione, and indirectly impairs GPX4 activity, thereby sensitizing tumor cells to lipid peroxidation (41, 60, 61). Pharmacologic glutathione depletion, for example with buthionine sulfoximine, similarly weakens antioxidant buffering capacity and may be useful where direct GPX4 inhibition is not yet tractable (61, 62). GPX4-independent defense systems are also emerging as therapeutically relevant resistance nodes. The FSP1-CoQ pathway can provide a major route of escape from ferroptosis, particularly in tumors with strong baseline antioxidant adaptation, whereas GCH1-BH4 and mitochondrial defense programs may contribute to persistent buffering in parallel (30–33, 63–65).

Recent lipid biology broadens the therapeutic landscape beyond canonical GPX4-centric approaches. Because MBOAT1/2 can suppress ferroptosis independently of GPX4 and FSP1, sex hormone-driven tumors may be sensitized by combining estrogen- or androgen-receptor blockade with ferroptosis induction (7). Likewise, modulation of distal cholesterol biosynthesis alters 7-DHC-mediated radical trapping and therefore influences ferroptosis sensitivity in a strongly context-dependent manner (8–10). These mechanisms are not yet clinically mature, but they illustrate how biomarker-guided targeting of lipid-remodeling pathways may complement direct antioxidant blockade.

A complementary therapeutic approach is to amplify iron-dependent lipid damage directly. FIN56 and FINO2 exemplify this strategy, promoting ferroptosis through mechanisms that include GPX4 destabilization, CoQ depletion, and iron-mediated lipid oxidation (66–68). In principle, such agents may be particularly effective in tumors already close to the ferroptotic threshold because of polyunsaturated membrane enrichment, PUFA-ePL accumulation, or elevated basal oxidative stress. Collectively, these strategies support a context-matched therapeutic model in which ferroptosis induction is guided by tumor lipid composition, antioxidant redundancy, and therapy-adapted cell-state vulnerabilities. This framework also provides a rationale for combining ferroptosis-targeted agents with chemotherapy, radiotherapy, or immunotherapy in settings where oxidative stress and metabolic adaptation are already prominent.

## 4 Tumor and cell-state contexts with ferroptosis vulnerabilities

Ferroptosis susceptibility is unlikely to be uniform across cancers because it is shaped by both relatively stable tumor-context features and dynamic changes in cellular state. To distinguish tumor-intrinsic context from adaptive plasticity, **Table 2A** summarizes representative lineage- and genotype-associated ferroptosis vulnerability contexts, whereas **Table 2B** highlights adaptive cell states that may create transient but therapeutically exploitable ferroptosis dependencies. These tables are intended as a conceptual framework rather than an exhaustive ranking, and the strength of supporting evidence varies across contexts.

**Table 2A:** Representative tumor lineage- and genotype-associated ferroptosis vulnerability contexts in cancer.

Tumor context	Baseline ferroptosis positioning	Dominant metabolic/redox feature	Key vulnerability node	Therapeutic rationale	Candidate biomarkers	Key references
Pancreatic ductal adenocarcinoma	Near-threshold, ferroptosis-prone	KRAS-associated oxidative stress and cystine dependence	system xC−/glutathione/GPX4 axis	Cysteine depletion; system xC− inhibition; GPX4 targeting	KRAS mutation; high SLC7A11; elevated glutathione; ACSL4	(43, 69)
KEAP1-mutant NSCLC/LUAD	Ferroptosis-resistant	NRF2-driven antioxidant buffering	GPX4/FSP1-CoQ defenses	Dual antioxidant targeting; radiotherapy combinations; ferroptosis sensitization	KEAP1 mutation; NRF2 signature; FSP1 expression; SLC7A11	(36–38, 63, 70)
Triple-negative breast cancer	Ferroptosis-sensitive	PUFA-enriched membrane lipid metabolism	ACSL4-PUFA axis	Lipid peroxidation induction; GPX4 inhibition	ACSL4; mesenchymal signature; elevated ROS	(13, 45, 46)
Clear-cell carcinomas (renal/ovarian)	GPX4-dependent, ferroptosis-prone state	HIF-linked PUFA enrichment and clear-cell lipidome	GPX4-centered detoxification system	Direct GPX4 targeting; context-matched combination strategies	Clear-cell morphology; HIF-2α/HILPDA; PUFA-rich lipidome	(47)
Glioblastoma	Stress-conditioned sensitivity with strong antioxidant dependence	High cystine uptake and redox adaptation	system xC−/glutathione axis	Cystine restriction; oxidative stress amplification; radiotherapy combinations	SLC7A11 expression; therapy-resistant phenotype	(48, 71, 72)
Hepatocellular carcinoma	Suggested/adaptive ferroptosis response	Lipid peroxidation with compensatory antioxidant and sterol buffering	GPX4/redox buffering; sterol metabolism	Sorafenib sensitization; context-matched ferroptosis combinations	Lipid peroxidation markers; ACSL4; sterol-synthesis context	(10, 73)
Head and neck squamous cell carcinoma	Therapy-adapted resistance with targetable redox dependence	Glutathione metabolism dependence	Glutathione-GPX4 axis	Glutathione depletion; GPX4 targeting	Cisplatin resistance; elevated glutathione metabolism; SLC7A11	(74)
Ovarian cancer, platinum-resistant	Ferroptosis-resistant but targetable	NRF2-linked antioxidant adaptation	Glutathione-GPX4/system xC− axis	Glutathione depletion; system xC− inhibition; combination therapy	NRF2 activation; platinum resistance; SLC7A11; SCD1	(26, 55)

These lineage/genotype contexts are intended as representative rather than exhaustive, and the strength of evidence varies by tumor type. In particular, the hepatocellular carcinoma row should be interpreted cautiously as a context in which ferroptosis is plausible but not uniformly established.

**Table 2B:** Representative adaptive and plastic tumor cell states associated with ferroptosis vulnerability.

Cell state	Ferroptosis positioning	Dominant metabolic/redox feature	Key vulnerability node	Therapeutic rationale	Candidate biomarkers	Key references
Drug-tolerant persister cells	Near-threshold, highly vulnerable	Elevated ROS and dependence on lipid peroxide detoxification	GPX4-centered defense system	Direct GPX4 inhibition; combined antioxidant blockade	Elevated ROS; GPX4 dependency; therapy persistence markers	(45, 46)
EMT-like states	Ferroptosis-sensitive	PUFA-enriched membranes and lipid remodeling	ACSL4-PUFA axis	Lipid peroxidation induction; ferroptosis sensitization	ACSL4; PUFA enrichment; EMT markers	(13, 45)
Mesenchymal-like states	Ferroptosis-sensitive	Oxidative stress with oxidizable membrane lipids	GPX4/ACSL4-linked dependence	GPX4 inhibition; lipid peroxide amplification	Mesenchymal signature; ACSL4; elevated ROS	(13, 45, 46)
Cancer stem cell-like states	Context-dependent; often buffered	Stemness programs coupled to redox buffering and adaptive lipid remodeling	system xC−/glutathione/GPX4 and lipid-droplet programs	Context-matched sensitization rather than assumed vulnerability	Stemness markers; SLC7A11; glutathione-related programs	(48)

Adaptive cell states can generate transient ferroptosis liabilities, but dedifferentiation is not uniformly pro-ferroptotic. This table therefore distinguishes strongly sensitized persister/mesenchymal states from cancer stem cell-like states that may remain comparatively buffered depending on context.

Pancreatic ductal adenocarcinoma remains a paradigmatic example of a tumor type positioned close to the ferroptotic threshold. KRAS-driven metabolic stress in this disease is associated with pronounced cystine dependence and sensitivity to cysteine depletion, suggesting that disruption of the system xC−/glutathione/GPX4 axis may be particularly effective (43, 69). In contrast, lung adenocarcinoma and non-small cell lung cancer with KEAP1 inactivation often exhibit NRF2-driven antioxidant programs that enhance glutathione synthesis, lipid-peroxide detoxification, and ferroptosis resistance, making dual antioxidant blockade or FSP1-directed approaches more attractive (36–38, 63, 70). Triple-negative breast cancer often displays polyunsaturated membrane enrichment and elevated ACSL4 expression, features associated with heightened ferroptosis sensitivity (13, 45, 46). Clear-cell carcinomas of renal and ovarian origin provide another vulnerability context in which HIF-linked PUFA enrichment can create a GPX4-dependent state (47). Glioblastoma shows strong dependence on cystine import and redox buffering, providing a rationale for combining cystine restriction with radiotherapy-induced oxidative stress (48, 71, 72). Hepatocellular carcinoma can undergo ferroptosis in sorafenib-responsive models, but the evidence remains more heterogeneous and adaptive antioxidant rewiring or sterol metabolism may modify this response (10, 73). Therapy resistance can conversely generate ferroptosis-resistant states that nonetheless reveal targetable liabilities, as illustrated by cisplatin-resistant head and neck squamous cell carcinoma and platinum-resistant ovarian cancer, in which glutathione metabolism and NRF2-linked antioxidant adaptation can become dominant survival programs (26, 55, 74).

Beyond lineage-specific effects, ferroptosis vulnerability is strongly shaped by tumor cell plasticity. Drug-tolerant persister, epithelial-mesenchymal transition (EMT)-like, and mesenchymal-like states commonly exhibit elevated ROS, polyunsaturated membrane remodeling, and increased dependence on lipid peroxide detoxification (13, 45, 46). These features position such populations closer to the ferroptotic threshold and may create selective therapeutic windows. Importantly, dedifferentiation should not be treated as uniformly pro-ferroptotic: while therapy-adapted mesenchymal or persister states often become vulnerable, stemness-associated states can also deploy stronger redox buffering and lipid-remodeling defenses. Ferroptosis vulnerability should therefore be viewed as a context-dependent and state-dependent metabolic phenotype rather than a fixed binary property.

## 5 Biomarkers of ferroptosis susceptibility

Clinical translation of ferroptosis-based therapies will depend on robust biomarkers that can both predict tumor susceptibility and monitor pathway engagement during treatment. Unlike targeted therapies driven by a single genomic alteration, ferroptosis sensitivity reflects a multidimensional metabolic state shaped by membrane lipid composition, iron handling, cystine availability, antioxidant buffering, and cell state. No single biomarker is therefore likely to capture ferroptosis vulnerability across all tumor contexts.

One major biomarker class comprises genetic and transcriptional alterations affecting redox homeostasis. Activation of the KEAP1-NRF2 pathway is particularly relevant, as KEAP1 loss stabilizes NRF2 and induces antioxidant programs that promote cystine utilization, NADPH generation, and lipid-peroxide detoxification (36–38). In glioma, high SLC7A11 expression has been associated with aggressive behavior and stem-like properties, supporting its value as both a biomarker and a potential therapeutic target (48, 71). Oncogenic context adds another layer of stratification: RAS-driven tumors often experience elevated oxidative stress and may depend on cystine import and GPX4-mediated detoxification, whereas MYC-driven programs can enhance NADPH production and glutathione metabolism in selected settings and thereby suppress ferroptosis sensitivity (11, 35, 75–78). Expression of MBOAT1/2 or sex hormone receptor programs may identify tumors with GPX4-independent phospholipid-remodeling defenses (7).

Lipid metabolic features likely offer the strongest functional relevance. ACSL4 expression, which promotes incorporation of omega-6 polyunsaturated fatty acids into membrane phospholipids, correlates with ferroptosis sensitivity in multiple settings (13). Oxidized arachidonoyl- and adrenoyl-phosphatidylethanolamines remain canonical ferroptotic signals, while di-polyunsaturated phospholipids and PUFA-ePLs provide additional substrate-level readouts of membrane oxidative liability (5, 6, 15, 20). Distal sterol metabolism may also become informative: 7-DHC abundance or signatures of distal cholesterol biosynthesis could indicate a radical-trapping, ferroptosis-buffered state in specific tumors (8–10). These observations suggest that lipidomic profiling may provide a more direct functional readout of ferroptosis readiness than transcript-level markers alone.

**Figure 2:**
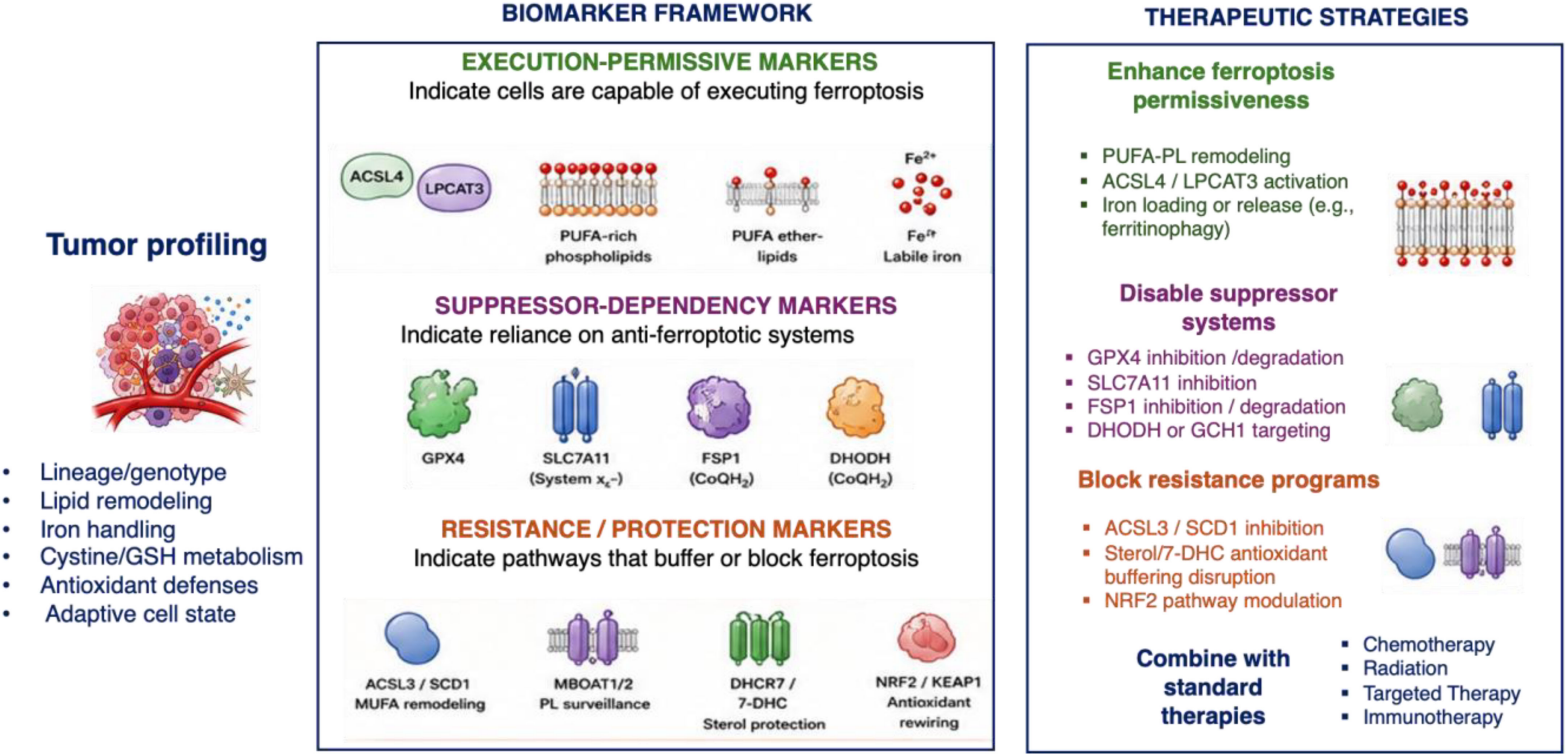
Biomarker-guided therapeutic deployment of ferroptosis-based strategies. Ferroptosis susceptibility reflects a multidimensional tumor state shaped by lipid architecture, iron handling, cystine/GSH metabolism, antioxidant buffering, lineage/genotype, and adaptive plasticity. Execution-permissive biomarkers, including ACSL4/LPCAT3 activity, PUFA-rich phospholipids, PUFA ether lipids, oxidized PUFA-phospholipids, and labile iron, identify tumors with increased lipid-peroxidation liability. Suppressor-dependency biomarkers, such as GPX4–GSH, SLC7A11/xC-, FSP1-2, DHODH–CoQH2 and GCH1–BH_4_ indicate antioxidant nodes that may be inhibited or depleted to lower the ferroptosis threshold. Resistance and protection markers, including ACSL3/SCD1-driven MUFA remodeling, MBOAT1/2-dependent phospholipid remodeling, DHCR7/7-DHC-associated sterol antioxidant buffering, and KEAP1/NRF2-driven antioxidant rewiring, may identify tumors requiring resistance-pathway blockade or rational combination therapy. This framework supports biomarker-matched use of ferroptosis inducers, suppressor-axis inhibitors or degraders, resistance-pathway modulators, and combinations with chemotherapy, radiotherapy, targeted therapy, or immunotherapy.

Taken together, ferroptosis vulnerability is best understood as a dynamic metabolic state rather than a fixed genetic trait. In practical terms, patient stratification will likely require integrated biomarker frameworks combining markers of oxidizable lipid architecture, antioxidant buffering capacity, tumor lineage, and adaptive cell state. Features such as ACSL4 expression, polyunsaturated lipid signatures, SLC7A11-dependent cystine utilization, glutathione-related programs, activity of parallel defense systems including FSP1 and GCH1, sterol/7-DHC remodeling, and state markers of mesenchymal transition or drug-tolerant persistence are likely to be more informative when interpreted collectively rather than in isolation. **Figure 2** summarizes this translational logic and illustrates how biomarker patterns may inform the choice between disabling buffering and amplifying lipid peroxidation.

## 6 Clinical challenges and future perspectives

Despite rapid progress in ferroptosis biology, translation into effective cancer therapy remains challenging because ferroptosis sensitivity is highly context dependent. Tumors exist along a continuum of ferroptotic liability defined by the balance between lipid peroxidation pressure and antioxidant defense capacity, and this balance varies across tumor types, genetic backgrounds, cell states, and microenvironmental conditions. Approaches that effectively induce ferroptosis in one setting may therefore fail in another unless matched to the relevant metabolic dependencies (2, 41).

A major obstacle is the redundancy and plasticity of antioxidant defense systems. Although GPX4 is a central ferroptosis suppressor, tumor cells can compensate through parallel pathways including FSP1-CoQ, GCH1-BH4, MBOAT1/2-mediated phospholipid remodeling, 7-DHC-dependent radical trapping, and mitochondrial programs that sustain NADPH-dependent redox homeostasis (7–10, 30–34). These overlapping networks limit the efficacy of single-node inhibition and argue for rational combination strategies capable of simultaneously disabling multiple layers of protection.

Drug development presents an additional challenge. Many ferroptosis inducers, including covalent GPX4 inhibitors, remain valuable chemical probes but are not yet clinically suitable because of pharmacokinetic limitations, off-target reactivity, and systemic toxicity (11, 53). To address these barriers, current efforts increasingly focus on targeted delivery systems, including nanoparticles, tumor-activated prodrugs, and antibody-drug conjugates designed to spatially restrict ferroptosis induction within tumors (79–82). The tumor immune microenvironment adds further complexity: ferroptotic tumor cells can release oxidized lipid mediators that influence dendritic cell activation and inflammatory signaling, yet ferroptosis can also compromise immune-cell viability, underscoring the need to integrate ferroptosis-targeted approaches thoughtfully with immunotherapy (50–52, 83–87).

Looking forward, advances in lipidomics, metabolomics, spatial profiling, and single-cell technologies are enabling increasingly high-resolution characterization of ferroptosis states in tumors (5, 8, 9, 20, 41). The next major step will be the development of robust biomarker frameworks capable of defining tumor lipid states *in vivo*, predicting ferroptosis susceptibility, and monitoring treatment engagement in patients. In practical terms, the most plausible future workflow is biomarker guided: identify tumors with high oxidizable-lipid burden and limited buffering for direct ferroptosis induction; identify tumors dominated by NRF2/SLC7A11/FSP1/MBOAT1/2/7-DHC defenses for rational combination blockade; and monitor treatment with integrated transcriptional and lipidomic readouts. Such a strategy is more realistic than treating ferroptosis as a universal anticancer modality.

## 7 Conclusion

Ferroptosis should not be viewed as a universal anticancer strategy, but rather as a context-dependent metabolic vulnerability that emerges under defined oncogenic, lipid, and microenvironmental conditions. Recent advances in lipid biology have made this point sharper: membrane composition, ether-phospholipid plasticity, 7-dehydrocholesterol metabolism, and GPX4-independent phospholipid-remodeling defenses all help determine whether a cancer cell remains buffered or becomes vulnerable to oxidative collapse. By integrating these mechanisms with tumor lineage, adaptive state, and biomarker development, ferroptosis research is moving from descriptive cell-death biology toward a more precise oncology framework. The most promising path forward will likely depend on identifying tumor states in which membrane lipid architecture, antioxidant redundancy, and therapy-induced plasticity converge to create selective liabilities that can be matched to the appropriate ferroptosis-based intervention.

## Glossary

7-DHC: 7-dehydrocholesterol
ACSL4: acyl-CoA synthetase long-chain family member 4
ADCs: antibody-drug conjugates
AGPS: alkylglycerone phosphate synthase
AKT: protein kinase B
APR-246: PRIMA-1Met
BH4: tetrahydrobiopterin
BSO: buthionine sulfoximine
CD8+: cluster of differentiation 8-positive
CoQ: coenzyme Q
DGAT1/2: diacylglycerol acyltransferase 1/2
DHODH: dihydroorotate dehydrogenase
EMT: epithelial-mesenchymal transition
FAR1: fatty acyl-CoA reductase 1
FIN56: ferroptosis inducer 56
FINO2: ferroptosis inducer 2
FSP1: ferroptosis suppressor protein 1
GCH1: GTP cyclohydrolase 1
GPX4: glutathione peroxidase 4
HIF: hypoxia-inducible factor
KEAP1: Kelch-like ECH-associated protein 1
KRAS: Kirsten rat sarcoma viral oncogene homolog
LOX: lipoxygenase
LPCAT3: lysophosphatidylcholine acyltransferase 3
LUAD: lung adenocarcinoma
MAPK: mitogen-activated protein kinase
MBOAT1/2: membrane-bound O-acyltransferase domain-containing 1/2
mTOR: mechanistic target of rapamycin
MUFA: monounsaturated fatty acid
MYC: MYC proto-oncogene
NADPH: nicotinamide adenine dinucleotide phosphate, reduced form
NRF2: nuclear factor erythroid 2-related factor 2
NSCLC: non-small cell lung cancer
PE: phosphatidylethanolamine
PEBP1: phosphatidylethanolamine-binding protein 1
PI3K: phosphoinositide 3-kinase
PKCβII: protein kinase C beta II
PROTAC: proteolysis-targeting chimera
PUFA: polyunsaturated fatty acid
PUFA-ePLs: polyunsaturated ether phospholipids
RAS: rat sarcoma family of small GTPases
RAF: rapidly accelerated fibrosarcoma kinase
ROS: reactive oxygen species
SCD1: stearoyl-CoA desaturase 1
SLC7A11: solute carrier family 7 member 11
system xC−: cystine/glutamate antiporter
TMEM189: transmembrane protein 189
TNBC: triple-negative breast cancer
TP53: tumor protein p53
xCT: light-chain subunit of system xC−
